# The Efficacy of Trunk Bracing With an Instrumented Corrective Exercise on Spinal Deformity, Pulmonary Function, Trunk Muscle Endurance, and Quality of Life in Adolescent Idiopathic Scoliosis: Protocol for a Parallel Groups Clinical Study

**DOI:** 10.2196/43265

**Published:** 2023-03-29

**Authors:** Zeinab Rezaeian, Ali Andalib, Fateme Bokaee, Maryam Poorpooneh Najafabadi, Gillian Yeowell, Ebrahim Sadeghi-Demneh

**Affiliations:** 1 Department of Orthotics and Prosthetics, Musculoskeletal Research Center, School of Rehabilitation Sciences, Isfahan University of Medical Sciences Isfahan Iran; 2 Department of Orthopaedic Surgery, Musculoskeletal Research Center, School of Medicine, Isfahan University of Medical Sciences Isfahan Iran; 3 Department of Physiotherapy, Musculoskeletal Research Center School of Rehabilitation Sciences Isfahan University of Medical Sciences Isfahan Iran; 4 Iranian Red Crescent Society, Rehabilitation Center of Esfahan Esfahan Iran; 5 Department of Health Professions, Manchester Metropolitan University Manchester United Kingdom; 6 Department of Orthotics and Prosthetics, Musculoskeletal Research Center School of Rehabilitation Sciences Isfahan University of Medical Sciences Isfahan Iran

**Keywords:** home exercise, orthotic device, quality of life, scoliosis

## Abstract

**Background:**

Adolescent idiopathic scoliosis is a 3D spine distortion with an unidentified etiology. It results in noticeable trunk deformity, decreased muscle strength and endurance at the trunk, changes in chest volume, breathing issues, and ultimately a decline in the quality of life. Trunk bracing and corrective exercises make up most of the treatment of patients with scoliosis when their deformity is between 20° and 45°, and they have not yet attained skeletal maturity. Evidence suggests that spinal deformity in people with scoliosis may result from improper motor control. Automatic response training is an exercise therapy technique that can modify the pattern of trunk muscle control for supporting the spinal column in normal alignment. An apparatus called a cantilever device is required for this type of exercise, which facilitates training at home. In spite of research showing the benefit of braces and therapeutic exercise in adolescents with scoliosis, less emphasis has been given to the impact of home-based training, especially when this intervention is paired with braces.

**Objective:**

We aim to compare the efficacy of bracing and a conventional exercise program to a combination treatment that includes trunk bracing and exercises with a cantilever device performed at home on the degree of spine curvature, pulmonary function, trunk muscular endurance, and quality of life.

**Methods:**

This study was a 2-arms parallel-group clinical study. A total of 16 adolescents with idiopathic scoliosis and single lumbar and thoracolumbar curves of 20°-45° were recruited and randomly assigned into 2 groups. Group A received a combination of trunk bracing and exercise using an instrument known as a “cantilever.” Group B (controls) received trunk bracing and a conventional exercise program (without a tool). The study outcomes were the Cobb angle of the scoliotic curve, pulmonary function, the endurance of the trunk muscles, and quality of life. The study outcomes were measured at 2 time points: before the intervention (T1) and 12 weeks following the start of the intervention (T2; at this time, the intervention period has been completed). Multivariate analysis of variance was used to test between- and within-group differences.

**Results:**

Recruitment for this study began in fall 2022 and is expected to be completed by the end of summer 2023.

**Conclusions:**

We studied the efficacy of a combined trunk bracing program and postural response exercises using a cantilever device in treating adolescent idiopathic scoliosis and compared it with trunk bracing and conventional home exercises. Exercises performed at home using a cantilever device are anticipated to raise the endurance of trunk muscles, which will help reduce trunk deformity, enhance pulmonary function, and improve the quality of life of participants.

**Trial Registration:**

Iranian Registry of Clinical Trials IRCT20220330054371N1; https://www.irct.ir/trial/62811

**International Registered Report Identifier (IRRID):**

PRR1-10.2196/43265

## Introduction

Adolescent idiopathic scoliosis (AIS) is a 3D spine deformity with an unknown etiology [1]. It is stated that 0.47%-5.2% of people aged between 11 and 18 years have an AIS [2]. Typical side effects of scoliosis include apparent trunk deformities, pulmonary restriction, muscular weakness, activity limitations, and a decline in health-related quality of life [3,4]. The current treatment goals for AIS include preventing the progression of the deformity, resolving the scoliosis curve, and improving quality of life [5]. Treatment options for AIS vary depending on the degree of spinal curvature and skeletal growth [1]. Physiotherapy, exercise, and monitoring techniques are often used with patients with curves less than 20°, especially those with substantial remaining skeletal growth [6]. Trunk bracing and exercises are prescribed for curves between 20° and 45° to promote soft tissue flexibility and enhance the functions of muscles that support the trunk [6,7]. Surgical correction is a typical treatment for curves greater than 45° [6].

A major concern with AIS is the loss of motor control, which can manifest as asymmetrical or weak muscles [8]. Also, abnormal breathing patterns are observed in those with AIS, which can exacerbate the scoliosis deformity [7,9]. People with scoliosis frequently have less strength and endurance in their trunk muscles than people without the condition [10]. Therefore, exercise therapy is often regarded as the cornerstone of recommended treatment for AIS [11].

Trunk bracing is the most prevalent nonoperative method for preventing curve progression in mild to moderate AIS (ranging from 20° to 45° Cobb angles) [12]. Evidence suggests that the effectiveness varies with different brace types [13]. Rigid braces have shown superior effects in preventing curve progression than nonrigid braces [13,14]. The external corrective forces delivered via a rigid brace reduce asymmetric spine loading and thereby prevent asymmetric growth [15]. The rigid bracing can limit the user’s physical activity, resulting in a loss of muscular strength and endurance over time [16,17]. The long-term use of a stiff brace may impede rib cage expansion and decrease lung capacity [18,19]. The study agrees that using selective exercise can compensate for the adverse effects of rigid trunk bracing on muscular and respiratory functions [16,20-22]. Activation of the muscles involved in spinal stabilization can be beneficial in reducing the risk of treatment failure in AIS [23]. Providing expanding chambers in conjunction with pressure zones is a feature of innovative brace designs like the Cheneau that help to activate muscles inside the brace [24].

Scoliosis treatment guidelines recommend home exercise as an adjunct to brace therapy [22]. Physical training enhances the effects of the brace by increasing muscular strength and endurance and removing the drawbacks of trunk bracing as a single treatment [21,25]. Moreover, exercise reduces the curve’s progression and improves pulmonary function and the quality of life of people with AIS [21]. Recent ideas on conservative scoliosis therapy suggest that therapeutic exercise should be specifically tailored to modify the motor control of the spine [21]. It has been substantiated that the brain and spine do not communicate effectively in AIS [21,26]. The incorrect perception of gravity could affect muscle tone and cause spinal curvatures [26]. Postural response training is thought to improve the activation of spinal muscles and balance the body against external forces [5,21,26]. An apparatus known as a “cantilever” is typically needed for postural response training to apply external forces to the body during repeated exercise [26]. According to prior research, there is little study on the efficiency of combination brace and exercise therapy in AIS [13,22]. Furthermore, a thorough search of the literature by the authors also discovered that no prior research has yet evaluated the effectiveness of postural response training paired with trunk bracing. This study compares the efficacy of combination bracing and conventional exercise with combined bracing and postural response training. It has been hypothesized that postural control training will improve the functioning of the spine’s muscles, which will help to straighten the spine, increase chest space, enhance respiratory function, and eventually enhance an individual’s quality of life.

## Methods

### Study Design

This prospective study was a 2-armed, participant-blinded, randomized controlled trial (intervention vs control group) performed to investigate the objectives. The study procedure was reported using the TIDieR (Template for Intervention Description and Replication) checklist and associated guidelines. The Intervention Description and Replication checklist outlines a consistent method for meticulously recording information and enables authors to define treatments precisely in their research [27]. The CONSORT (Consolidated Standards of Reporting Trials) checklist was used to report data effectively in a clinical trial [28].

### Setting

Participants were consecutively recruited from individuals with AIS, Risser sign 0-2, and Cobb curvature 20°-45°, and were referred to a spine specialist (author AA) for their first evaluation in the Orthopedic Clinic at Al-Zahra Hospital in Isfahan, Iran.

### Sample Size

A total of 16 adolescents with single lumbar and thoracolumbar curves of 20°-45° and idiopathic scoliosis were recruited for the study. Based on the findings of an earlier study, the ideal sample size was calculated [29]. The Cobb angle was reported as 24.26° (SD 1.96°) for the intervention group and 26.59° (SD 3.57°) for the control group, respectively [29]. We calculated the sample size using G*power software (version 3.1.9.4, Universität Düsseldorf) and determined that 7 subjects in each trial arm would provide 60% power once α was set at .1. We also considered a 10% overall dropout rate (eg, lost to follow-up). Consequently, we aimed to recruit 16 individuals (8 in each study arm).

### Participants

A simple (convenient) sampling method was used to recruit the AIS individuals with single lumbar and thoracolumbar curves of 20°-45° at the orthopedic clinic. Physical assessments and spine x-rays taken by an orthopedic specialist were used to confirm the diagnosis of AIS [30]. The following are the inclusion requirements of the study: Risser sign (0-2) [31], a single moderate scoliosis curve (20°-45°) in the lumbar or thoracolumbar regions [32], physical and mental capacity to use braces and exercise equipment [33], the absence of AIS treatment in the preceding year [34], and physician approval to take part in the study. The exclusion criteria are as follows: deformities in the lower extremities [35]; having neuromuscular conditions like cerebral palsy, spina bifida, muscular dystrophy, congenital myopathy, and any other illness that prevents them from exercising [33]; and people with any history of surgery, trauma, fractures, or other spine injuries [33,35]. The parent of each eligible participant signed an informed consent form before the study. Participants were allowed to withdraw from the study at any time.

### Assignment, Randomization, and Blinding (Masking) Procedures

After verification of eligibility, participants were randomly assigned to one of 2 groups: (1) the intervention group (combined brace and home-based exercise aid; n=8) or (2) the control group (brace and scoliosis exercise program at home; n=8). Block randomization was used, 4 blocks with a block size of 4, to achieve balance in allocating participants to study arms [36]. The assessor uses Random Allocation Software for the randomization process (version 1.0) [37]. This study is single-blinded, so the measurement of the curve angle was undertaken by blinded assessors. The assessors measured the curve angle without being aware of the group assignment or individual information.

### Adverse Events and Dropouts

Adverse events were acknowledged and documented throughout the study to ensure participant safety. The final report summarized adverse event information, including the date and the participant’s experience. The details of research participants who dropped out were recorded. Furthermore, the causes of dropout, including COVID-19 symptoms, loss of follow-up, and illness, were disclosed.

### Study Arms and Content

**Figure 1:**
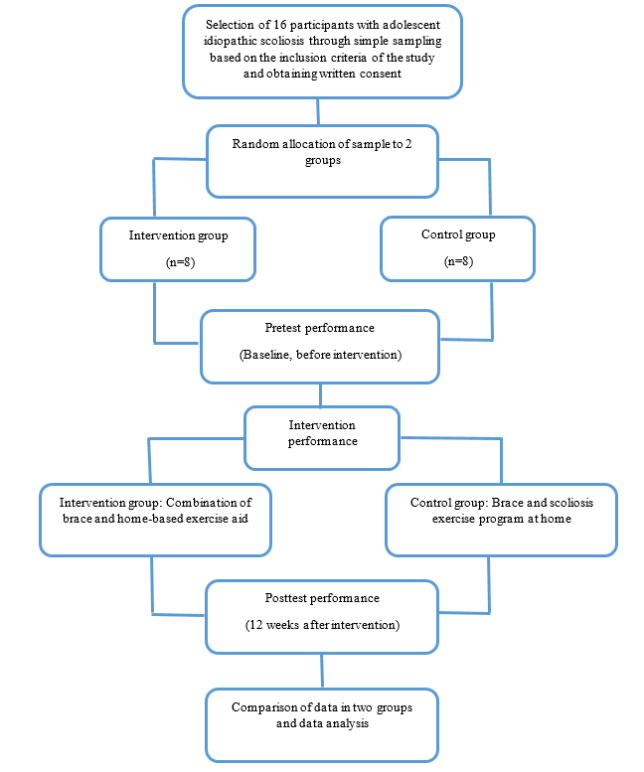
The study flowchart for the randomized controlled trial.

There were 2 study arms. For the intervention group, the first arm received a combination of brace and home-based postural response training. For the control group, the second arm received a brace and a traditional scoliosis exercise program at home. The outcomes include Cobb’s angle of curvature, pulmonary function, the endurance of the trunk muscles, and quality of life index. The study outcomes were measured at 2 time points: baseline, before the intervention (pretest), and 12 weeks after the intervention began [38] (posttest; at this time, the intervention period has been completed). Figure 1 presents the study process.

### Intervention

#### Cheneau Brace

**Figure 2:**
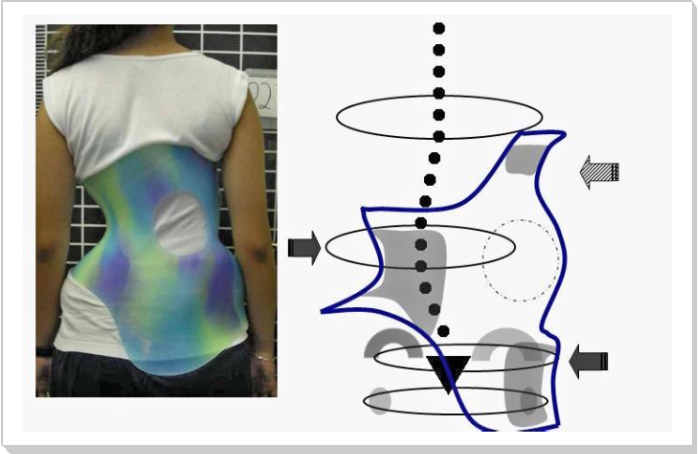
The E-type Cheneau brace for a single lumbar-thoracolumbar curve pattern [40].

Cheneau is a rigid trunk brace used to treat AIS [24]. This thoracolumbosacral brace is made of polypropylene with an anterior opening (Figure 2). The Cheneau brace applies pressure on the convexity of the curve and shifts the spine to the normal alignment. The brace walls allow the overcorrection of the curvature; thereby, the user can perform in-brace muscle contraction and therapeutic exercise [24,39]. In this study, all braces are single lumbar and thoracolumbar, also called E1 and E2, respectively [40]. The E-type Cheneau braces for single lumbar or thoracolumbar curves (Figure 2) are custom-molded orthoses that were fabricated by a qualified orthotist. The brace users were instructed how to wear it and asked to wear it full-time during the trial course.

#### Cantilever Device

**Figure 3:**
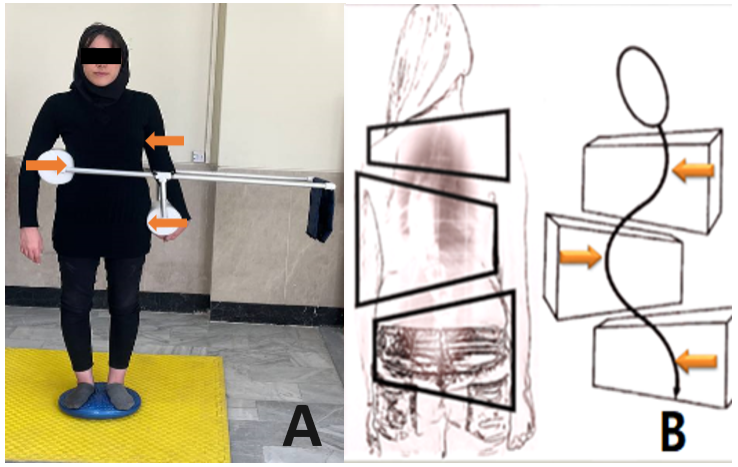
(A) The cantilever device and (B) the 3-point system for correction of deformity [41].

In this study, postural response training for individuals with AIS was carried out using a torso trainer device known as the “cantilever” (Figure 3). The cantilever device increases the mechanical advantage of the corrective forces applied to the trunk for curve correction [26]. The 3 pressure points that make up the system’s correcting mechanism (Figure 3A) provide transverse force to the spine in order to reduce deformity [26]. Each cantilever device was customized by an orthotist to fit the size of each unique user and has a robust tubular structure (as shown in Figure 3A). Low-density polyurethane was used for the device’s pads. The thoracic pad of the device was fitted on the convex side of the deformity, while the lumbar pad is located between the lower ribs and the iliac crest. To enhance the resistance during an incremental training program, up to 4-pound weights can be applied at the end of the lever.

#### Postural Response Training

This exercise is to modify the activity pattern of the muscles to stabilize the spine. It has been reported that response training could enhance the role of proprioceptive feedback in detecting spinal misalignment [26]. Therefore, postural response training is expected to help regulate the appropriate muscle activity in people with AIS [26,42]. There are 3 phases in each session for postural response [42] as outlined in Textbox 1.

**Three phases of postural response training.**

- Participants will first attempt to slide their hand down their outside leg, on the convex side of the curvature while standing. After holding this flexed position for 15 seconds, they will return to the starting position. Participants will complete this workout 20 times with a 15-second break between each repetition.
- Participants will carefully put on the cantilever device during the second phase, placing the pressure pads where the therapist had instructed them and adjusting the length of the levers and the weight distribution. Then participants will stand on a vestibular disk and attempt to adjust their spine toward the convexity of the curve by using a cantilever device so that the weight hanging on the device is at the horizontal level. After holding this level position for 15 seconds, they will return to the starting position and rest for 15 seconds. Participants will perform this workout 10 times, followed by a 5-minute break. This process will be repeated 4 times so that the total training time of the second phase lasts 40 minutes.
- In the last stage, the participants kneel on the floor with their buttocks resting on the heels and stretch their arms as far forward as they can. They hold this posture for 15 seconds.

#### Conventional Scoliosis Exercise

The exercise outlined in Textbox 2 was instructed to each participant and carried out under a physiotherapist’s supervision. These exercises are designed based on the literature on exercise for AIS [43,44].

**Steps of conventional scoliosis exercise.**

- The participant will face backward on a chair. Sitting far back, they hold hands on the back of the chair, elbows sideways and upwards. The knee on the convex side will be held further back for pelvic derotation. The head and trunk will lean to the convex side.
- The participant is in side-lying on the concave side with the arm stretched underneath the head.
- The participant kneels on the floor with thighs vertical and arms extended forward in a V-position, then brings the upper chest toward the floor.
- The participant will place on all 4 limbs. The participant performs a breathing exercise and simultaneously rotates the trunk to correct the scoliotic curve.
- The participant will be placed in the lateral side-lying position so that the concave side faces the floor. The elbow will be bent, and the arm will support body’s weight. The participant then tries to lift the pelvis and correct the spinal curve.
- Participants will sit on a chair with their backs on the wall. Participants will keep their feet together, arms stretched, elbows straight, and hands touching the wall from behind. They lean forward with their trunk as far as possible, without losing the contact between their hands and the wall.

### Outcome Measures

The study outcome measures were calculated throughout the course of 2 sessions. All tests were conducted with a certified orthotic practitioner before intervention (baseline) and after 12 weeks of follow-up required to complete the intervention (posttest).

### Primary Outcome Measures

#### Spinal Deformity

**Figure 4:**
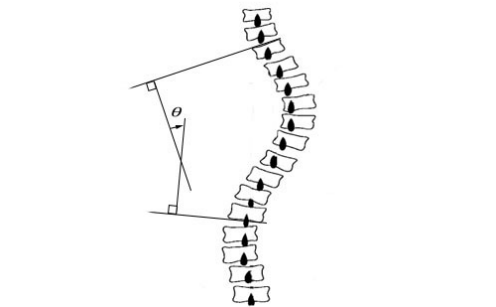
Measurement of the Cobb angle (φ).

The lateral deviation of the spine in the frontal plane was measured using the Cobb angle. This method is the gold standard for correctly identifying, assessing, and predicting the progression of the scoliosis curve [30,45]. An orthopedic specialist manually measured the Cobb angle using an anteroposterior spine x-ray. The Cobb angle was measured by drawing an end-point line at the upper and lower end of the scoliosis vertebrae. The Cobb angle (Figure 4) is the intersection of the 2 vertical lines drawn on each end-point line [30].

### Secondary Outcome Measures

#### Pulmonary Function

A digital spirometer (HI-301, Chestgraph, Japan) was used to evaluate pulmonary function. The spirometer was used to measure the following parameters: forced vital capacity, peak expiratory flow, forced expiratory volume in 1 second, vital capacity expiratory, vital capacity inspiratory, total lung capacity, and vital capacity. The participants were instructed to take off the brace at least 2 hours before the test. Spirometry is conducted while the patient is seated in a chair [46]. Each participant was asked to do a regular inspiration first. The participants took a deep exhalation after the mouthpiece of the gadget was put in his mouth. The experiment ended once the participant has had a deep inspiration, a deep expiration, and a deep inspiration. Care was taken during the test to keep the mouth and the device’s input together. The time between her inspiration and exhalation should not be broken, and a thorough exhalation should continue for at least 6 seconds [46]. This test was measured 3 times, and the average values were reported for data analysis. Each participant was given 2 minutes of resting time between trials. All tests were administered at the same time during the day in a quiet location, and participants were asked to refrain from engaging in strenuous exercise prior to testing.

#### The Trunk Muscles’ Endurance

The lumbar trunk muscle endurance test was used to measure trunk flexor and extensor endurance, while the side-bridge test measured lateral trunk flexor endurance.

**Figure 5:**
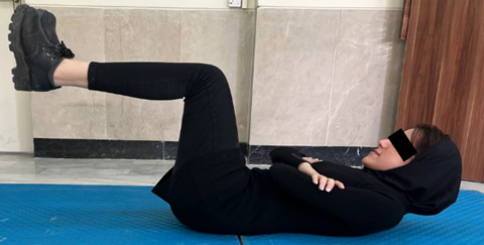
Endurance test of trunk flexor muscles.

**Figure 6:**
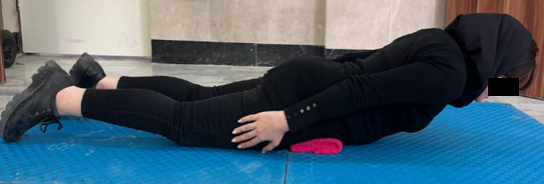
Endurance test of trunk extensor muscles.

The next assessment was carried out to evaluate the patient’s flexor muscle endurance [47]. Each participant was instructed to lie on a flat surface in the supine position and elevate their lower limbs off the ground while maintaining a 90° flexion angle in both the hip and knee joints. Then participant strives to remove both scapulae from the flat surface by maintaining maximal neck flexion as their arms are positioned crosswise on their chest (Figure 5). The patient was instructed to lie prone on a flat surface, elevate the sternum from the surface, and maintain maximal neck flexion to test the patient’s trunk extensor muscle endurance [47]. To lessen lumbar lordosis, a cushion was positioned beneath the lower abdomen (Figure 6).

**Figure 7:**
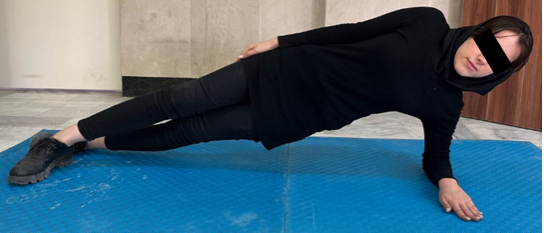
Endurance test of trunk lateral flexor muscles.

Next, the participants were requested to lie on their lateral side to assess the strength of the lateral muscles in the trunk. The individual rises the upper body and pelvis straight from the surface, while the forearm and leg bear the body’s weight (Figure 7). The participant keeps this position as long as possible, and the examiner records the time before the pelvic drops [48]. This test was repeated for the opposite side, and a 5-minute rest was given between 2 trials [48].

#### Quality of Life

Quality of life was measured using the Persian version of the Italian Spine Youth Quality of life [49]. This questionnaire has good validity and reliability (intraclass correlation coefficient ≥0.7) to use for people with AIS [49]. This questionnaire includes 13 spinal health–related questions and was filled out by each participant. The responses to each question were quantified based on a 3-point rating scale (0-2) with 3 possible replies (never, sometimes, often). The quality of life ranged from 0 to 26, with 0 being the best quality of life [50].

#### Demographic and Clinical Data

Age; weight; height; BMI; and the type, location, degree, and magnitude of the scoliosis curve as well as the Risser sign, were prospectively gathered and noted on report forms.

### Data Analysis

Analysis of covariance was used to compare how well each treatment influenced the outcomes of the study. Trunk bracing with postural response training versus bracing with regular exercise is the independent variable, and the dependent variables are study outcomes recorded after the exercise protocols were completed. In these statistical analyses, the covariant is the baseline measurement of the research results among the individuals. The preliminary statistics were carried out to ensure that the assumptions of normality, linearity, homogeneity of variances, homogeneity of regression slopes, and accurate measurement of covariates are not violated. Statistical analyses were conducted using the SPSS (version 19; IBM Corp). The level of significance was set at .05 for all tests.

### Ethics Approval

The Isfahan University of Medical Sciences Regional Ethics Committee approved the study protocol (IR.MUI.NUREMA.REC.1401.025). Then, the research protocol was registered in the Iranian Registry of Clinical Trials on May 9, 2022 (IRCT20220330054371N1).

## Results

Recruitment for this study began in fall 2022 and is expected to be completed by the end of summer 2023.

## Discussion

### Overview

It is well acknowledged that expected scoliosis consequences include dysfunction of motor control, which could demonstrate asymmetrical or weak muscles [8], less muscular strength and endurance [10], and abnormal breathing patterns [7,9]. Consequently, exercise therapy and conditioning are consistently indicated as a necessary element of AIS treatments [11].

Trunk bracing is the most popular nonoperative technique to prevent curve progression in mild to moderate AIS (from 20° to 45° Cobb angle) [12]. Evidence, however, indicates that rigid bracing might restrict a user’s physical activity, which can lead to a loss of muscle strength and endurance [17]. Besides, rigid trunk bracing could impede rib cage expansion and reduce lung capacity [19]. Exercise can mitigate the negative impact of rigid trunk bracing on muscular and respiratory functions [20-22]. The study agrees that specific exercise can counteract the adverse effects of rigid trunk bracing on the respiratory and muscular functioning of people with AIS [20,21].

Therapeutic exercise appears necessary as part of conservative scoliosis treatment focusing on changing the spine’s alignment control [21]. An incorrect perception of gravity can affect muscle tone and cause spinal curvatures [26]. Postural response training aims to change how one feels gravity and how active one’s trunk muscles are. Future research is necessary because there is little research that supports the effectiveness of this approach.

### Strengths and Limitations

The study’s most noteworthy strength is the combination of trunk bracing and exercise in the treatment of AIS. There is limited research on the benefits of trunk bracing with exercise therapy [13,22]. The Cheneau brace and postural response training were developed using an active strategy for curvature correction [26]. According to this strategy, individuals with AIS should practice aggressively using their trunk muscles to keep their spine in a normal alignment [44]. Compliance with the intervention may be one of the most significant difficulties in the present study. Through phone calls or SMS text messages, authors strived to keep track of each participant’s commitment to brace wearing and exercise and record that information in the logbook.

## Appendix

Multimedia Appendix 1 Peer review report from Isfahan University of Medical Sciences - Vice Chancellery for Research and Technology (Isfahan, Iran).

## Abbreviations

AIS: adolescent idiopathic scoliosis.
CONSORT: Consolidated Standards of Reporting Trials
TIDieR: Template for Intervention Description and Replication

## Data Availability

The data sets generated or analyzed during this study are available from the corresponding author upon reasonable request.
