# Supplementary material for: The Efficacy of Trunk Bracing With an Instrumented Corrective Exercise on Spinal Deformity, Pulmonary Function, Trunk Muscle Endurance, and Quality of Life in Adolescent Idiopathic Scoliosis: Protocol for a Parallel Groups Clinical Study
Source: JMIR Res Protoc. 2023 Mar 29;12:e43265. doi: 10.2196/43265 (PMC10131677; doi:10.2196/43265)
Supplement: Multimedia Appendix 1 [file resprot_v12i1e43265_app1.pdf]

Isfahan University of Medical Sciences
Vice Chancellery for Research and Technology

Research Grant Peer review Form

**Funding Source**
Postgraduate studies and research program
**Amount**
327,000,000 Rials
Electronic Submission Code
34001028
Principle investigator
Ebrahim Sadeghi Demneh
Co-investigators
Zeinab Rezaeian, Fateme Bokaee, Ali Andalib, Maryam Poorponeh, Gillian Yeowell
Project title
The efficacy of trunk bracing with an instrumented corrective exercise on spinal deformity, pulmonary function, trunk muscle endurance and quality of life in adolescents idiopathic scoliosis: Protocol for a parallel-groups clinical study

Dear Ebrahim Sadeghi Demneh

Thank you for submitting your manuscript to the university's vice chancellory for research and technology. We have received comments from the reviewers on your proposal. Reviewers have included some feedback on the different sections of the manuscript and hope you find these comments helpful.

Your research proposal should become acceptable for registration, pending suitable revisions and modifications of the proposal in light of the appended reviewer comments. Note that it may still be necessary to receive feedback from the reviewers prior to making a final decision.

When resubmitting your manuscript, please carefully consider all issues mentioned in the reviewers' comments, outline every change made the point by point, and provide suitable rebuttals for any comments not addressed.

To submit your revised manuscript go to https://researches.mui.ac.ir/index.phtml and log in as an academic member, where you will see a menu item called 'Proposal Needing Revision'.

Please resubmit your manuscript by Jan 26, 2022.

Reviewers' comments:

Introduction

- Please add related articles on the cheneau brace and cantilever device.
- It is clear that the two interventions of brace and cantilever device have a positive history in studies, therefore, it should be clarified that what is the necessity of plan performance?
- Please define the necessity of examining the respiratory function in adolescent idiopathic scoliosis.
- It appears that the covid-19 pandemic is not the main concern in this research, so it is suggested to eliminate this cause.

Method

- Please mention the importance of the curvature type, apex location and pain existence in inclusion and exclusion criteria.
- Explain how to evaluate acceptance and duration of using brace and cantilever device in patients.
- Please add the characteristics of the spirometer and some details about the person using it.
- The method of performing and evaluating exercise should be explained. Add a physiotherapist consultant to this research.
- Explain the action mechanism of the cantilever device. Please mention device's purpose is to strengthen which group of trunk muscles.
- Add the guidelines related to the duration of the follow-up period.
- Please explain the endurance tests of the trunk lateral flexor muscles. The relation of the trunk muscles with the cantilever device should be mentioned.
- Mention how to eliminate the effect of Cobb angle measurement error.
- Consider the rigidity of the pressure pads and the amount of applied force of the cantilever device.
- Please explain the efficacy of the cantilever device on the endurance of the trunk muscles.
